# Trends in Premiums, Claims, and Enrollment for Fully Insured Large Group, Small Group, and Individual Health Plans From 2011 to 2021

**DOI:** 10.1001/jamanetworkopen.2023.8791

**Published:** 2023-04-18

**Authors:** Elizabeth Plummer, Allison Percy, Ge Bai

**Affiliations:** 1 Neeley School of Business, Texas Christian University, Fort Worth; 2 Burnett School of Medicine at TCU, Fort Worth, Texas; 3 Health Analysis Division, Congressional Budget Office, Washington, DC; 4 Johns Hopkins Carey Business School, Baltimore, Maryland; 5 Johns Hopkins Bloomberg School of Public Health, Baltimore, Maryland

## Abstract

This economic evaluation compares trends in premiums, claims, and enrollment among fully insured large group, small group, and individual health plans in the US from 2001 to 2021.

## Introduction

In 2021, more than 20% of the US population was covered by fully insured large group, small group, and individual private health insurance plans.^1^ These plans are purchased from insurers by large employers, small employers, and individuals, respectively. In contrast, in self-insured plans, employers bear the risk for covered health care claims of employees and dependents enrolled in plans. Prior research for fully insured group plans is scarce, while studies of individual plans have shown rising premiums and fluctuating enrollment.^2,3,4^ This study compares the 11-year trends (2011-2021) in premiums, claims, and enrollment across fully insured large group, small group, and individual plans nationwide and for each state.

## Methods

This economic evaluation followed the CHEERS reporting guideline. This study did not fulfill criteria for human participants research in accordance with 45 CFR §46. The Patient Protection and Affordable Care Act (ACA) requires that private health insurers submit annual reports for each state and market (large group, small group, individual) in which they do business.^1^ Large group plans are generally purchased by firms with more than 50 full-time equivalent (FTE) employees, small group plans are purchased by firms with 50 or fewer FTE employees, and individual plans are purchased through ACA exchanges or off-exchange. (Beginning in 2016, California, Colorado, New York, and Vermont expanded the definition of small group to include firms with up to 100 FTE employees.) Reports include insurers’ annual premiums, claims, and enrollment (including dependents; measured as life-years with partial-year enrollment factored in). The sample included all large group, small group, and individual plan-years that reported positive premiums, claims, and enrollment from 2011 through 2021 (all available years), covering 67 to 79 million enrollees each year.

We examined nationwide median annual premium and claims per enrollee and total enrollment in each market. We calculated the values for these variables in 2021 and the corresponding percentage change from 2011. All dollar amounts were adjusted to 2021 values using the medical Consumer Price Index.^5^ SAS, version 9.4 was used for analysis.

## Results

From 2011 to 2021, median annual premium per enrollee grew by 5.9% ($5701 to $6035) for large group plans, 9.6% ($5683 to $6228) for small group plans, and 59.0% ($3574 to $5683) for individual plans (Figure, A). Median claims per enrollee grew faster by 14.9% for large group plans, 21.0% for small group plans, and 96.6% for individual plans (Figure, B). Large and small group plans had a decline in total enrollment from 49.4 to 41.1 million and from 18.8 to 11.5 million, respectively, while individual plan enrollment increased from 10.9 to 14.9 million (Figure, C).

**Figure.**
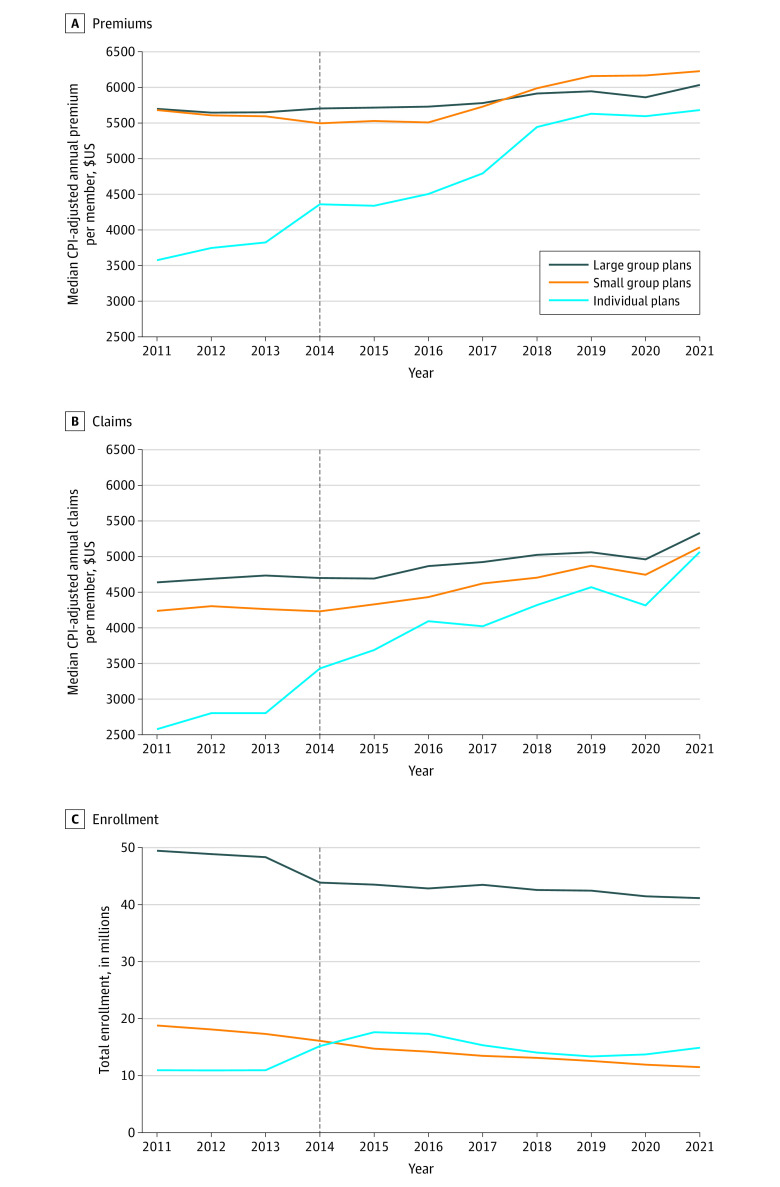
Median Annual Premiums, Claims, and Enrollment by Market From 2011 to 2021 The vertical dashed lines indicate when several major provisions, including insurance exchanges and premium subsidies, became effective in 2014. All dollar amounts were adjusted to 2021 values using the medical Consumer Price Index (CPI).^5^ A, Annual premiums per enrollee were measured as a plan’s annual premiums divided by number of life-years (including dependents). The test of the 2011 to 2021 difference in median annual premiums for each of the 3 markets was statistically significant (*P* < .001). B, Annual claims per enrollee were measured as a plan’s annual claims divided by number of life-years (including dependents). The test of the 2011 to 2021 difference in median annual claims for each of the three markets was statistically significant (*P* < .001). C, Total enrollment was measured by life-years (including dependents). Life-years was calculated as the total number of lives (including dependents) insured on a prespecified day of each month divided by 12, which takes into account partial-year enrollment.

Across all markets, median annual premiums and claims per enrollee increased for almost every state (Table). Large group enrollment decreased in 38 states and Washington, DC; small group enrollment decreased in every state except Idaho; and individual enrollment increased in 30 states and Washington, DC.

**Table.** Median Annual Premium, Claims, and Enrollment for 2021 by State and Market^a^

State	Median, $ (% change from 2011)						Total enrollment, in 1000 (% change from 2011)		
	Annual premium per enrollee			Annual claims per enrollee			Large group	Small group	Individual
	Large group	Small group	Individual	Large group	Small group	Individual			
Alaska	9301 (15.8)	7757 (−7.2)	7115 (66.5)	8097 (11.8)	6336 (−3.0)	424 (−86.3)	74 (53.4)	13 (−60.5)	20 (24.4)
Alabama	4694 (0.9)	4444 (−15.3)	3918 (15.9)	4582 (7.2)	4149 (27.8)	2696 (91.9)	583 (15.4)	191 (−38.1)	207 (23.1)
Arkansas	4943 (8.4)	4978 (−19.7)	5227 (37.9)	4292 (24.5)	3682 (7.3)	4575 (121.0)	237 (9.4)	88 (−33.1)	392 (242.4)
Arizona	5741 (5.7)	5862 (5.9)	5208 (43.9)	4934 (17.3)	4242 (14.0)	4752 (87.5)	429 (−31.7)	153 (−29.0)	197 (−24.5)
California	6234 (8.9)	5739 (4.1)	6118 (55.4)	5337 (15.5)	4901 (9.1)	5469 (62.8)	10 522 (−0.9)	2190 (−5.8)	2318 (42.2)
Colorado	6316 (5.9)	6267 (−0.8)	5030 (59.7)	5824 (20.1)	4679 (−0.8)	4284 (57.1)	590 (−15.8)	234 (−9.9)	240 (−18.3)
Connecticut	7542 (8.5)	6408 (3.7)	7665 (64.1)	6549 (14.6)	6577 (32.2)	8225 (136.8)	283 (−37.4)	102 (−61.7)	115 (3.7)
Washington, DC	6155 (2.3)	6231 (−12.1)	3440 (−8.7)	5444 (29.6)	5086 (28.5)	4305 (138.0)	619 (−27.5)	88 (−3.1)	18 (1.7)
Delaware	7675 (23.9)	7525 (2.2)	7805 (95.9)	7295 (55.0)	5347 (4.9)	7647 (199.3)	56 (−41.2)	23 (−52.9)	29 (51.9)
Florida	6059 (−0.1)	6654 (8.5)	5759 (50.0)	5396 (3.8)	5868 (29.2)	5484 (71.5)	2119 (4.5)	428 (−51.8)	2391 (198.2)
Georgia	5995 (9.9)	6862 (15.7)	5685 (66.5)	5667 (28.5)	5721 (25.1)	6822 (170.2)	969 (8.8)	124 (−76.7)	544 (59.9)
Hawaii	5683 (23.3)	5748 (13.7)	4696 (37.2)	4526 (14.3)	4780 (17.3)	2874 (34.4)	614 (16.3)	111 (−26.8)	35 (8.5)
Iowa	5773 (7.6)	5289 (21.9)	6890 (139.1)	5108 (17.6)	4816 (36.2)	4415 (122.9)	305 (−18.1)	142 (−21.7)	98 (−45.7)
Idaho	4896 (5.3)	4439 (−1.9)	5357 (107.2)	4365 (5.0)	3790 (15.2)	4366 (85.7)	197 (−14.1)	95 (6.2)	89 (−14.3)
Illinois	6148 (0.6)	6160 (7.0)	5171 (23.8)	5578 (9.7)	5417 (20.3)	4921 (66.8)	1815 (−15.2)	514 (−23.2)	379 (−18.0)
Indiana	6277 (−0.8)	6667 (24.7)	5689 (66.3)	5103 (10.0)	5990 (46.7)	5212 (114.7)	325 (−13.5)	123 (−67.7)	135 (−25.7)
Kansas	5696 (12.4)	5597 (3.5)	6663 (87.3)	5074 (23.8)	4382 (11.9)	5804 (171.3)	377 (−13.6)	121 (−36.0)	111 (−16.6)
Kentucky	5730 (9.9)	7066 (33.5)	5792 (137.7)	4842 (9.5)	4664 (20.5)	5352 (229.4)	326 (−12.4)	48 (−75.7)	92 (−35.3)
Louisiana	6254 (7.0)	6220 (4.7)	6794 (79.3)	5182 (5.8)	4974 (14.8)	3427 (33.0)	384 (16.2)	171 (−43.4)	115 (−35.7)
Massachusetts	7039 (2.4)	7038 (9.6)	6779 (21.3)	6220 (10.7)	6052 (12.8)	6236 (92.5)	1090 (−18.1)	390 (−36.8)	333 (235.0)
Maryland	6271 (4.4)	6105 (7.6)	4462 (48.0)	5467 (17.6)	4800 (2.8)	3685 (119.1)	991 (−6.2)	254 (−27.5)	238 (31.1)
Maine	6516 (−3.5)	7090 (8.3)	4071 (−18.1)	6061 (15.8)	5276 (14.8)	5032 (14.7)	177 (−9.3)	47 (−45.3)	64 (71.8)
Michigan	5467 (15.0)	5110 (9.0)	4382 (50.5)	4804 (9.9)	4310 (14.4)	3707 (83.5)	1388 (−34.7)	466 (−27.3)	341 (0.6)
Minnesota	5740 (−15.7)	5478 (2.7)	3625 (−1.4)	5415 (5.2)	4218 (−1.7)	4557 (20.2)	632 (−17.6)	160 (−53.4)	116 (−53.9)
Missouri	6063 (6.8)	6769 (31.7)	6566 (122.3)	5566 (22.0)	5042 (27.5)	5799 (153.2)	591 (−22.6)	133 (−60.6)	247 (−0.8)
Mississippi	5231 (6.4)	5332 (−5.3)	4467 (26.2)	4247 (29.8)	3718 (−9.0)	4448 (84.4)	199 (8.8)	85 (−38.7)	140 (78.0)
Montana	5950 (22.2)	6205 (5.5)	5748 (61.7)	5420 (31.2)	4865 (26.8)	5569 (94.6)	71 (−29.1)	46 (−12.0)	53 (0.8)
North Carolina	5790 (0.9)	5941 (1.4)	6472 (101.7)	4884 (25.8)	4616 (4.6)	4538 (116.9)	538 (−6.6)	292 (−21.3)	603 (43.4)
North Dakota	6077 (19.1)	5794 (28.2)	4565 (30.0)	5428 (21.3)	4956 (42.9)	5268 (99.4)	150 (5.5)	55 (−14.5)	44 (0.5)
Nebraska	5817 (9.4)	6968 (23.4)	5832 (70.3)	4791 (7.6)	5579 (38.9)	3458 (40.9)	218 (10.8)	36 (−66.9)	92 (−24.7)
New Hampshire	7547 (15.5)	6480 (−9.7)	5136 (16.6)	6282 (20.8)	5655 (−0.8)	3593 (15.5)	141 (−10.6)	63 (−33.3)	57 (60.4)
New Jersey	7871 (16.4)	7309 (17.2)	5783 (7.3)	6195 (15.3)	6812 (26.6)	4853 (−10.9)	771 (−29.5)	297 (−58.0)	360 (129.1)
New Mexico	5668 (3.0)	5641 (−9.1)	4697 (42.6)	5522 (23.2)	4850 (11.1)	3903 (76.8)	113 (−37.1)	48 (−20.0)	49 (−20.3)
Nevada	5398 (1.5)	5249 (−1.7)	5339 (32.9)	4705 (23.9)	4310 (14.0)	4508 (42.6)	374 (−4.4)	83 (−19.5)	109 (23.3)
New York	7650 (18.4)	6706 (16.1)	6598 (45.0)	6579 (20.3)	6237 (22.7)	7441 (52.8)	2310 (−64.8)	880 (−42.4)	317 (27.4)
Ohio	6005 (11.0)	7455 (43.2)	6313 (77.9)	5230 (15.1)	5841 (47.7)	5163 (99.1)	919 (−11.9)	204 (−78.2)	255 (−22.9)
Oklahoma	5917 (10.6)	5134 (−13.7)	5557 (61.0)	5488 (44.3)	4365 (7.8)	4928 (129.0)	383 (−29.6)	180 (−0.3)	179 (43.5)
Oregon	6278 (5.8)	5575 (3.1)	6532 (81.1)	5558 (9.2)	5033 (11.4)	5795 (90.9)	664 (−3.9)	179 (−22.7)	172 (−2.3)
Pennsylvania	6568 (15.2)	7332 (34.8)	5398 (58.8)	5368 (17.9)	5417 (30.6)	4373 (61.1)	1400 (−41.3)	506 (−51.7)	428 (−13.6)
Rhode Island	6101 (−2.7)	6532 (9.0)	4942 (6.2)	5346 (−1.9)	5354 (17.7)	3858 (6.2)	112 (−40.0)	48 (−47.8)	42 (169.5)
South Carolina	6169 (6.1)	7024 (13.9)	5292 (52.0)	5340 (28.8)	5394 (41.3)	4554 (106.2)	303 (−16.1)	76 (−55.4)	277 (113.5)
South Dakota	5970 (8.8)	6280 (10.5)	5909 (48.7)	5223 (1.8)	4814 (5.3)	5824 (103.5)	100 (−10.0)	49 (−13.1)	54 (−11.4)
Tennessee	5479 (3.1)	5829 (6.0)	6464 (67.3)	4159 (4.3)	4438 (11.2)	4276 (104.7)	524 (12.4)	185 (−51.0)	253 (2.9)
Texas	5418 (−3.2)	6744 (17.1)	5338 (33.9)	5029 (22.1)	4840 (12.2)	5467 (109.5)	2422 (34.2)	736 (−40.4)	1483 (106.5)
Utah	4721 (6.0)	4134 (8.5)	4093 (76.7)	4206 (12.0)	3493 (13.4)	3081 (57.4)	407 (−12.9)	159 (−35.9)	244 (76.3)
Virginia	5827 (−0.1)	6207 (13.8)	6566 (82.8)	4870 (−3.1)	4729 (22.2)	5134 (82.8)	1079 (−17.4)	311 (−34.3)	292 (−8.1)
Vermont	6756 (8.4)	6102 (−5.0)	6670 (253.7)	6412 (28.4)	6111 (32.5)	6879 (697.6)	28 (−61.6)	41 (−36.9)	31 (55.9)
Washington	5627 (−1.4)	5965 (11.7)	6239 (56.7)	5175 (9.2)	4978 (27.5)	5268 (87.5)	1064 (−13.6)	301 (−5.2)	247 (−15.8)
Wisconsin	6071 (−2.4)	6285 (11.9)	7701 (102.2)	5707 (6.1)	5646 (14.0)	6501 (144.0)	988 (−3.4)	178 (−57.1)	212 (18.0)
West Virginia	7153 (19.6)	7807 (14.2)	5908 (36.6)	6200 (21.9)	6615 (32.6)	4666 (102.2)	133 (−4.8)	30 (−55.9)	20 (−19.1)
Wyoming	6418 (0.2)	7185 (4.2)	6539 (88.4)	5031 (−11.6)	6639 (43.8)	4902 (90.8)	31 (−11.4)	15 (−43.7)	31 (30.2)
Median	6059 (6.4)	6220 (7.6)	5748 (56.7)	5346 (15.5)	4974 (15.2)	4853 (90.9)	407 (−12.9)	133 (−36.9)	172 (3.7)

^a^ All dollar amounts were adjusted to 2021 values using the medical Consumer Price Index.^5^

## Discussion

In this economic evaluation, Consumer Price Index–adjusted median annual premiums for individual plans grew faster than for group plans. Unlike group plans, individual plans purchased on-exchange allow eligible enrollees to receive ACA premium tax credits and cost-sharing reductions. Rising enrollment in individual plans may be associated with the availability of these subsidies and the ACA’s individual mandate and guaranteed issue requirements. Declining enrollment in fully insured group plans may reflect some dependents or employees with low income moving to other sources of coverage (including Medicaid and subsidized exchange plans) and factors such as premium increases and employment changes. Survey data have shown an increase in the share of small firm employees enrolled in self-insured plans, which could explain some of the decline in small group enrollment in our data.^6^ Trends in premiums, claims, and enrollment varied by state, possibly in association with socioeconomic factors and different state laws and regulations. This study was confined to fully insured plans and limited by lack of information on benefit design, pricing, or service utilization and potential reporting inaccuracies.

## Notes

Supplement.
Data Sharing Statement
