# Supplementary material for: Trends in Premiums, Claims, and Enrollment for Fully Insured Large Group, Small Group, and Individual Health Plans From 2011 to 2021
Source: JAMA Netw Open. 2023 Apr 18;6(4):e238791. doi: 10.1001/jamanetworkopen.2023.8791 (PMC10114028; doi:10.1001/jamanetworkopen.2023.8791)
Supplement: Supplement. — Data Sharing Statement [file jamanetwopen-e238791-s001.pdf]

Data Sharing Statement

Plummer. Trends in Premiums, Claims, and Enrollment for Fully Insured Large Group, Small Group, and Individual Health Plans From 2011 to 2021. *JAMA Netw Open*. Published April 18, 2023. doi:10.1001/jamanetworkopen.2023.8791

Data
Data available: No

Additional Information
Explanation for why data not available: The data used in this study is publicly available.
